# The Relationship between Youth Police Stops and Depression among Fathers

**DOI:** 10.1007/s11524-022-00713-8

**Published:** 2023-02-15

**Authors:** Kristin Turney

**Affiliations:** Department of Sociology, University of California—Irvine, 3151 Social Science Plaza, Irvine, CA 92697 USA

**Keywords:** Depression, Mental health, Policing, Youth

## Abstract

Research shows youth police contact—a stressor experienced by more than one-quarter of urban-born youth by age 15—has deleterious mental health consequences for both youth and their mothers. Less is known about how youth’s fathers respond to this police contact, despite differences in how men and women respond to stress and relate to their children. I use data from the Fragile Families and Child Wellbeing Study to investigate the association between youth police stops and depression among youth’s fathers. Results show that fathers of youth stopped by the police, compared to fathers of youth not stopped by the police, are more likely to report depression, net of father and youth characteristics associated with selection into experiencing youth police stops. This association is concentrated among non-Black fathers and fathers of girls. The findings highlight how the repercussions of youth criminal legal contact extend to youth’s fathers and, more broadly, suggest that future research incorporate the responses of men connected to those enduring criminal legal contact.

The widespread nature of the criminal legal system means that youth commonly experience police contact. More than one-fifth of people have experienced a police stop by early adulthood [1]. More than one-fourth of urban youth—and two-fifths of urban Black boys—report a police stop by age 15, with many of these youth reporting stops accompanied by intrusive behaviors such as frisks or searches [2]. Police contact is an adverse childhood experience that leads to schooling disruptions, increased delinquency and contact with the criminal legal system, and physical and mental health impairments [2–7]. Youth stopped by the police, compared to their counterparts, report more mental health problems including depressive symptoms, anxiety, and post-traumatic stress disorder [1, 8–11]. In addition, aligned with the life course perspective that highlights the interconnected nature of stressors [12, 13], research finds that mothers of youth stopped by the police experience mental health impairments [14, 15]. Additional types of criminal legal contact among youth, such as incarceration, are also associated with mental health impairments among their mothers [16, 17].

Despite accumulating research showing that criminal legal contact is a stressor with deleterious mental health consequences for both youth and their mothers, little is known about how youth’s fathers respond to police stops. On the one hand, the damaging ramifications of police stops may extend to youth’s fathers. Youth police stops—and the corresponding trauma, distress, and despair—may directly impair the mental health of these fathers. Youth police stops may also facilitate anticipatory stress among these fathers (for example, worry about future criminal legal contact or concern about the short- and long-term effects of the stop on their youth) or changes in the youth’s behavior [6, 11, 15], both of which may impair mental health among fathers. Youth’s fathers may have had their own criminal legal contact, given that men are more likely than women to experience criminal legal contact, and may especially experience distress given their knowledge of what a police stop can entail [18]. On the other hand, youth police contact may have no ramifications for fathers’ mental health, as there are sex differences in how men and women respond to stress [19, 20], relate to their children [21], and exhibit mental health struggles [22, 23]. Alternatively, given the unequal exposure to youth police stops across race and youth sex, fathers’ responses to stressors may vary across these demographic groups [24].

I use data from the Fragile Families and Child Wellbeing Study (FFCWB), a cohort of youth born in urban areas around the turn of the twenty-first century and followed for 15 years, to investigate the association between youth police stops and mental health among youth’s fathers. I focus on depression, a commonly experienced mental health condition that is consequential for both youth and fathers’ well-being [25, 26]. I first examine the average association between youth police stops and fathers’ depression, net of characteristics associated with youth’s exposure to police contact and fathers’ history of depression. I then examine variation in the association between youth police stops and fathers’ depression by race (comparing Black and non-Black fathers) and youth sex (comparing fathers of boys to fathers of girls). The results show that depression is more common among fathers of youth stopped by the police than among fathers of youth not stopped by the police. The results also show that these associations are concentrated among non-Black fathers and fathers of girls. Taken together, the findings highlight that stressors such as youth police stops proliferate to youth’s fathers and, by generally excluding how this stressor proliferates to fathers, existing research excludes the full range of spillover consequences of criminal legal contact.

## Method

### Data

I estimate the association between youth police stops and fathers’ depression using data from the Fragile Families and Child Wellbeing Study, a cohort of nearly 5000 children born in urban areas around the turn of the twenty-first century and followed through adolescence [27]. Children’s biological mothers and fathers were interviewed as soon as possible after children were born (most often in the hospital). They were re-interviewed five additional times over a 15-year period (when children were approximately 1, 3, 5, 9, and 15 years old). Children and their primary caregivers (commonly their mother but occasionally their father or another adult) were interviewed at the 15-year survey. About three-fifths of respondents eligible for study participation (including 77% of primary caregivers and 74% of youth) participated in the 15-year survey. This paper’s analytic sample includes the 252 fathers who were interviewed as children’s primary caregivers at the 15-year survey. Fathers are considered primary caregivers if the youth lived with them at least half-time and lived with their biological mother for less than half-time.

### Measures

#### Fathers’ Depression

The measure of fathers’ depression comes from fathers’ responses to the Composite International Diagnostic Interview Short Form (CIDI-SF) at the 15-year survey [28]. Fathers were asked whether, at some time during the past year, they had feelings of depression or were unable to enjoy normally pleasurable things. Fathers who reported experiencing at least one of these two conditions most of the day and every day for a 2-week period were asked an additional seven questions (about losing interest in things, feeling tired, experiencing a change in weight of at least 10 pounds, having trouble sleeping, having trouble concentrating, feeling worthless, and thinking about death). Fathers who answered affirmatively to three or more of these seven questions are considered to have experienced depression in the past year. About 11.51% of fathers experienced depression in the past year.

#### Youth Police Stops

A binary variable indicates the father reports, at the 15-year survey, that the youth had experienced a police stop while on the street, at school, in a car, or some other place. Nearly one-fifth (18.25%) of fathers report the youth had been stopped by the police, which is lower than the frequency of youth who report a police stop themselves (32.54%). I use fathers’ reports of police stops as many of the proposed pathways linking youth police stops to fathers’ depression are contingent on fathers’ knowledge of the stop.

#### Control Variables

The multivariable analyses adjust for father and youth characteristics to isolate the association between youth police stops and fathers’ depression, important given that youth police stops are not randomly distributed across the population. All control variables are measured prior to the measure of youth police stops. Fathers’ demographic characteristics include race/ethnicity (non-Hispanic White, non-Hispanic Black, Hispanic, non-Hispanic other race), immigrant status (1 = *born outside the USA*), and relationship status with the youth’s mother (married, cohabiting, non-residential). Fathers’ socioeconomic status is measured by material hardship (a sum of 11 types of deprivation such as eviction and food insecurity). Fathers’ mental health includes heavy drinking (1 = *had four or more drinks in one sitting in the past month*) and drug use (1 = *used illicit drugs, including prescription drugs not prescribed, in the past month*). Fathers’ involvement with the criminal legal system is measured with two binary variables that indicate arrest (1 = *arrest history*) and incarceration (1 = *incarceration history*). Some analyses adjust for a lagged indicator of fathers’ depression (measured at the 9-year survey).

Youth characteristics include sex at birth (1 = *boy*), age, delinquency (a sum of responses to 17 statements including “paint graffiti or signs on someone else’s property or in a public place,” 1 = *yes*, 0 = *no*), and impulsivity (measured similar to fathers’ impulsivity). Some models adjust for additional youth characteristics measured at the 15-year survey (and therefore potentially endogenous to youth police stops) that may be especially associated with youth police stops. These characteristics include delinquency (an average of responses to 13 statements including “deliberately damaged property that did not belong to you,” 1 = *never* to 4 = *five or more times*), peer delinquency (an average of responses to 11 statements including “friend deliberately damaged property that did not belong to them,” 1 = *never* to 3 = *often*), going to a school with a stationed police officer, smoking history, drinking history, drug use history, arrest history, and incarceration history.

### Analytic Strategy

The analyses occur in three stages.

First, I estimate descriptive statistics (frequencies/means and standard deviations) of all father and youth characteristics for two groups: (1) fathers who report the youth experienced a police stop and (2) fathers who report the youth did not experience a police stop. I test for statistically significant differences across groups with chi-square tests or *t*-tests (depending on the distribution of the variable).

Second, I use linear probability models to estimate the average association between youth police stops and fathers’ depression. I use linear probability models for ease of interpretation, but logistic regression models produce estimates that are substantively similar to the linear probability models. The first model is unadjusted. The second model adjusts for father and youth characteristics. This model establishes proper time-ordering between fathers’ depression (measured at the 15-year survey), youth police stops (measured at the 15-year survey but almost exclusively occurring between the 9- and 15-year surveys), and the control variables (measured at or prior to the 9-year survey). Six youth in the analytic sample reported their first police stop at age 8, and findings are robust to excluding these youth. The third model adjusts for a lagged indicator of fathers’ depression (measured at the 9-year survey), which estimates the association between youth police stops and fathers’ depression net of fathers’ history of depression. The fourth model includes youth characteristics that are potentially endogenous to police stops (such as peer delinquency or drug use). This final model provides a conservative estimate of the association between youth police stops and fathers’ depression. Diagnostic checks suggest that multicollinearity does not bias the results (as the Variance Inflation Factor for all models is less than 2). All models include city fixed-effects to account for city-level variation in policing.

Third, I use linear probability models to estimate variation in the association between youth police stops and fathers’ depression by fathers’ race and youth’s sex at birth. I first estimate subgroup models for Black fathers and non-Black fathers, collapsing all non-Black fathers due to small sample sizes, and test for statistically significant differences across groups [29]. I then estimate subgroup models for boys and girls, again testing for statistically significant differences across groups. These models adjust for the control variables described above.

Most observations have complete data. Covariates are missing data for, on average, 10% of observations. I preserve missing data by producing 20 imputed data sets and pooling results across these data sets.

### Sample Description

Table 1 presents descriptive statistics of the analytic sample. Nearly half (44.35%) of fathers identify as non-Hispanic Black, with most other fathers identifying as Hispanic (26.67%) or non-Hispanic White (25.79%). About one-seventh (14.25%) of fathers were born outside the USA. Contact with the criminal legal system was common among fathers, as nearly two-thirds (63.47%) had an arrest history and nearly half (44.84%) had an incarceration history.

**Table 1:** Descriptive statistics of variables, for full sample and by fathers’ reports of youth police stops

	% or *M*	(S. D.)
Key variables		
Father depression (y15)	11.51%	
Youth stopped by police (y15)	18.25%	
Father characteristics		
Race/ethnicity (b)		
White (non-Hispanic)	25.79%	
Black (non-Hispanic)	44.35%	
Hispanic	26.67%	
Other race (non-Hispanic)	3.19%	
Foreign-born (b)	14.25%	
Relationship with adolescent’s mother (y9)		
Married	28.95%	
Cohabiting	7.18%	
Not residential	63.87%	
Educational attainment (y9)		
Less than high school	15.32%	
High school diploma or GED	25.28%	
More than high school	59.40%	
Material hardship (y9)	1.30	(1.99)
Heavy drinking (y9)	29.62%	
Illicit drug use (y9)	8.81%	
Ever stopped by police (y3, y5, y9)	63.47%	
Ever incarcerated (y1, y3, y5, y9)	44.84%	
Depression, lagged (y9)	13.71%	
Youth characteristics		
Male (b)	58.73%	
Age (y15)	15.72	(0.87)
Delinquency (y9)	1.00	(1.67)
Impulsivity (y15)	2.54	(0.71)
Delinquency (y15)	1.12	(0.21)
Peer delinquency (y15)	1.21	(0.31)
Police officer regularly stationed at school (y15)	80.95%	
Ever smoked (y15)	6.23%	
Ever drank alcohol without parents (y15)	22.82%	
Ever used marijuana or other drug (y15)	23.93%	
Ever arrested (y15)	8.73%	
Ever incarcerated (y15)	5.16%	
*N*	252	

*b* measured at baseline, *y1* measured at 1-year survey, *y3* measured at 3-year survey, *y5* measured at 5-year survey, *y9* measured at 9-year survey, *y15* measured at 15-year survey

## Results

Table 2 presents the characteristics of two groups of fathers—those who report the youth experienced a police stop and those who report the youth did not experience a police stop—and tests for statistically significant differences between these two groups. Depression is nearly three times as common among fathers of youth stopped by the police than among fathers of youth not stopped by the police (23.91% compared to 8.74%, *p* < 0.01). There are additional differences in fathers’ characteristics across these two groups. Fathers of youth stopped by the police, compared to other fathers, report more material hardship (1.82 compared to 1.18, *p* < 0.01). They are similarly likely to have been stopped by the police themselves (62.61% compared to 63.67%, *n*.*s*.) but are more likely to have an incarceration history (60.87% compared to 41.26%, *p* < 0.05). They are also more likely to have a history of depression (20.38% compared to 12.22%, *p* < 0.01). There are no differences in demographic characteristics (e.g., race/ethnicity, relationship status) between fathers of youth who are and are not stopped by the police.

**Table 2:** Descriptive statistics of variables, by fathers’ reports of youth police stops

	Police stop		No police stop		
	% or *M*	(S. D.)	% or *M*	(S. D.)	
Key variables					
Father depression	23.91%		8.74%		**
Youth stopped by police	–		–		
Father characteristics					
Race/ethnicity					
White (non-Hispanic)	21.74%		26.70%		
Black (non-Hispanic)	51.63%		42.72%		
Hispanic	24.35%		27.18%		
Other race (non-Hispanic)	2.28%		3.40%		
Foreign-born	10.54%		15.07%		
Relationship with adolescent’s mother					
Married	27.07%		29.37%		
Cohabiting	5.22%		7.62%		
Not residential	67.72%		63.01%		
Educational attainment					
Less than high school	15.98%		15.17%		
High school diploma or GED	29.46%		24.34%		
More than high school	54.57%		60.49%		
Material hardship	1.82	(2.53)	1.18	(1.82)	**
Heavy drinking	33.48%		28.76%		
Illicit drug use	7.17%		9.17%		
Ever stopped by police	62.61%		63.67%		
Ever incarcerated	60.87%		41.26%		*
Depression, lagged	20.38%		12.22%		**
Youth characteristics					
Male	67.39%		56.80%		
Age	15.81	(0.92)	15.69	(0.86)	
Delinquency	1.80	(2.59)	0.82	(1.32)	***
Impulsivity	2.72	(0.67)	2.50	(0.71)	^
Delinquency	1.21	(0.27)	1.10	(0.19)	**
Peer delinquency	1.33	(0.37)	1.19	(0.29)	**
Police officer regularly stationed at school	90.33%		78.86%		*
Ever smoked	22.50%		2.60%		***
Ever drank alcohol without parents	34.24%		20.27%		*
Ever used marijuana or other drug	45.33%		19.15%		***
Ever arrested	34.78%		2.91%		***
Ever incarcerated	21.74%		1.46%		***
*N*	46		206		

Asterisks indicate statistically significant differences between fathers of youth who experience a police stop and fathers of youth who do not experience a police stop. ^*p* < 0.10, *p < 0.05, **p < 0.01, ***p < 0.001

Table 2 also shows that youth stopped by the police are different than youth who are not stopped by the police. Youth stopped by the police, compared to their counterparts, report more delinquency at the 9-year survey (1.80 compared to 0.82, *p* < 0.001), more delinquency at the 15-year survey (1.21 compared to 1.10, *p* < 0.01), and more peer delinquency at the 15-year survey (1.33 compared to 1.19, *p* < 0.01). Youth stopped by the police are more likely to report having a police officer stationed at their school (90.33% compared to 78.86%, *p* < 0.05). They are also nine times more likely to report smoking (22.50% compared to 2.60%, *p* < 0.001), two times more likely to report alcohol use (34.24% compared to 20.27%, *p* < 0.05), and two times more likely to report drug use (45.33% compared to 19.15%, *p* < 0.001). They are also more likely to have experienced other criminal legal contact, with 34.78% and 21.74% of those stopped by the police reporting an arrest and incarceration, respectively, by the 15-year survey.

Table 3 presents results from the linear probability models that estimate the association between youth police stops and fathers’ depression. Model 1, the unadjusted association, shows a positive association between youth police stops and fathers’ depression (*b* = 0.15, *p* < 0.05). This positive association remains in model 2, which adjusts for all control variables (*b* = 0.15, *p* < 0.05); in model 3, which further adjusts for father’s history of depression (*b* = 0.15, *p* < 0.05); and in model 4, which further adjusts for youth characteristics that may be endogenous to police stops (*b* = 0.18, *p* < 0.05). This final model shows that an increase in youth police stops is associated with an increase in fathers’ depression, net of characteristics associated with selection into police stops. Importantly, youth police stops are the only statistically significant predictor of fathers’ depression across all models (and the magnitude of this coefficient is larger than the coefficients of nearly all control variables, including those possibly endogenous to youth police stops).

**Table 3:** Linear probability models estimating fathers’ depression as a function of youth police stops

	Model 1			Model 2			Model 3			Model 4		
	*Bivariate*			+ *controls*			+ *lagged depression*			+ *15-year youth characteristics*		
	*b*	(S. E.)		*b*	(S. E.)		*b*	(S. E.)		*b*	(S. E.)	
Youth stopped by police	0.15	(0.07)	*	0.15	(0.07)	*	0.15	(0.07)	*	0.18	(0.08)	*
Father characteristics												
Race/ethnicity (reference = White [non-Hispanic])												
Black (non-Hispanic)				− 0.09	(0.08)		− 0.09	(0.08)		− 0.08	(0.06)	
Hispanic				− 0.09	(0.07)		− 0.08	(0.07)		− 0.08	(0.07)	
Other race (non-Hispanic)				0.07	(0.20)		0.08	(0.20)		0.04	(0.16)	
Foreign-born				0.01	(0.07)		0.01	(0.07)		0.01	(0.08)	
Relationship with adolescent's father (reference = married)												
Cohabiting				− 0.13	(0.08)		− 0.11	(0.08)		− 0.11	(0.07)	
Not residential				− 0.07	(0.07)		− 0.07	(0.07)		− 0.07	(0.07)	
Educational attainment (reference = less than high school)												
High school diploma or GED				0.02	(0.07)		0.01	(0.07)		0.02	(0.07)	
More than high school				0.02	(0.06)		0.02	(0.06)		0.02	(0.05)	
Material hardship				0.00	(0.01)		0.00	(0.01)		0.00	(0.02)	
Heavy drinking				− 0.03	(0.05)		− 0.04	(0.06)		− 0.04	(0.06)	
Illicit drug use				0.08	(0.01)		0.07	(0.08)		0.08	(0.07)	
Ever stopped by police				0.07	(0.05)		0.07	(0.05)		0.07	(0.06)	
Ever incarcerated				0.02	(0.03)		0.02	(0.03)		0.02	(0.03)	
Depression, lagged							0.12	(0.08)		0.11	(0.08)	
Youth characteristics												
Male				0.03	(0.05)		0.02	(0.03)		0.02	(0.05)	
Age				− 0.03	(0.03)		− 0.04	(0.02)		− 0.03	(0.03)	
Delinquency (y9)				0.00	(0.02)		0.00	(0.03)		0.00	(0.02)	
Impulsivity				0.01	(0.03)		0.01	(0.08)		0.00	(0.03)	
Delinquency (y15)										0.23	(0.19)	
Peer delinquency										− 0.07	(0.10)	
Police officer regularly stationed at school										− 0.03	(0.06)	
Ever smoked										0.14	(0.17)	
Ever drank alcohol without parents										− 0.01	(0.07)	
Ever used marijuana or other drug										0.00	(0.06)	
Ever arrested										0.00	(0.11)	
Ever incarcerated										− 0.29	(0.13)	
Adjusted *R*-squared	0.03			0.10			0.11			0.16		
Constant	0.09			0.49			0.64			0.48		
*N*	252			252			252			252		

*y9* measured at 9-year survey, *y15* measured at 15-year survey. All models include city fixed-effects. ^*p* < 0.10, *p < 0.05, **p < 0.01, ***p < 0.001

The linear probability models are limited because they do not account for unobserved characteristics of fathers or youth that may render the association between youth police stops and fathers’ depression spurious. One way to strengthen causal inference is to estimate placebo models that use the explanatory variable (in this case, youth police stops) to estimate the outcome variable (in this case, fathers’ depression) at an earlier time point. Results from these supplemental analyses show no association between youth police stops and fathers’ depression at the 9-year survey (*b* = 0.03, *p* = 0.722), suggesting that unobserved characteristics do not threaten the main findings.

Table 4 presents results from linear probability models that examine variation in the association between youth police stops and fathers’ depression. The first two models consider variation by fathers’ race. There is an association between youth police stops and depression among non-Black fathers (*b* = 0.18, *p* < 0.10) but not among Black fathers (and, in fact, the coefficient for this group goes in the opposite direction [*b* =  − 0.05, *n*.*s*.]). A test for equality of coefficients across these groups shows the differences in coefficients are statistically significant (*p* < 0.01). The second two models consider variation by youth sex. These models show that the association between youth police stops and depression is larger for fathers of girls than fathers of boys. Neither of these subgroup associations reach statistical significance, and the coefficients across groups are not statistically different from one another.

**Table 4:** Linear probability models estimating fathers’ depression as a function of youth police stops, considering variation by race and sex

	Race						Sex				
	Black fathers			Non-Black fathers			Boys			Girls	
	*b*	(S. E.)		*b*	(S. E.)		*b*	(S. E.)		*b*	(S. E.)
Youth stopped by police	− 0.05	(0.05)		0.18	(0.10)	^	0.01	(0.06)		0.12	(0.08)
Father characteristics											
Race/ethnicity (reference = White [non-Hispanic])											
Black (non-Hispanic)	–	–		–	–		− 0.07	(0.10)		− 0.11	(0.09)
Hispanic	–	–		− 0.09	(0.08)		− 0.09	(0.12)		− 0.04	(0.11)
Other race (non-Hispanic)	–	–		0.02	(0.16)		− 0.20	(0.10)	^	0.50	(0.31)
Foreign-born	0.00	(0.14)		0.03	(0.08)		0.04	(0.10)		0.00	(0.10)
Relationship with adolescent's father (reference = married)											
Cohabiting	− 0.16	(0.15)		− 0.12	(0.10)		− 0.14	(0.08)		− 0.02	(0.15)
Not residential	− 0.09	(0.10)		− 0.05	(0.10)		− 0.03	(0.09)		− 0.12	(0.11)
Educational attainment (reference = less than high school)											
High school diploma or GED	− 0.03	(0.12)		− 0.01	(0.08)		0.01	(0.10)		0.04	(0.10)
More than high school	− 0.03	(0.10)		− 0.01	(0.08)		0.01	(0.10)		0.02	(0.09)
Material hardship	0.02	(0.02)		− 0.01	(0.01)		0.00	(0.01)		0.01	(0.02)
Heavy drinking	− 0.05	(0.08)		0.02	(0.08)		− 0.01	(0.07)		− 0.04	(0.09)
Illicit drug use	0.22	(0.09)	*	− 0.07	(0.14)		0.13	(0.11)		0.06	(0.12)
Ever stopped by police	0.06	(0.06)		0.10	(0.08)		0.04	(0.07)		0.09	(0.06)
Ever incarcerated	− 0.01	(0.06)		0.07	(0.06)		0.01	(0.06)		0.07	(0.06)
Youth characteristics											
Male	0.07	(0.06)		− 0.02	(0.06)		–	–		–	–
Age	− 0.02	(0.05)		− 0.03	(0.04)		− 0.03	(0.04)		− 0.04	(0.04)
Delinquency	0.02	(0.02)		0.01	(0.03)		0.01	(0.02)		0.01	(0.04)
Impulsivity	− 0.02	(0.03)		0.05	(0.06)		0.06	(0.05)		− 0.05	(0.03)
Adjusted *R*-squared	0.16			0.13			0.07			0.26	
Constant	0.35			0.44			0.48			0.76	
*N*	111–112			140–141			148			104	

Ns for race subgroups vary slightly across multiple imputed data sets. All models include city fixed-effects. *p < 0.05

## Discussion

The stress process perspective—with its focus on the unequal distribution of stressors across the population, the proliferation of stressors from the person experiencing the stressor to those connected to them, and the negative consequences of proliferating stressors for health—suggests that police stops may impair health among youth and those connected to them [13, 30]. Indeed, research documents the deleterious consequences of youth police stops for youth themselves and for their mothers [9–11, 14, 15, 31]. Less is known about whether and under what conditions these deleterious mental health consequences extend to youth’s fathers, despite good reasons to expect that fathers may respond differently than mothers. In this paper, I used data from the Fragile Families and Child Wellbeing Study, a population-based data source that includes information about both youth police stops and fathers’ mental health, to examine changes in fathers’ depression following a youth police stop. The analyses reveal two key findings.

First, results show that police stops among youth are positively associated with fathers’ depression. This association is robust to models that adjust for characteristics of fathers and youth that are associated with both youth police stops and fathers’ depression. The association is also robust to adjusting for fathers’ history of depression, which allows for an estimation of change in depression following the police stop, and to adjusting for potentially endogenous youth characteristics. These results are consistent with expectations from the stress process perspective that highlights how stressors can proliferate from the individual initially exposed to the stressor to those connected to them [29]. These findings are also consistent with research showing that youth police stops increase the probability of depression among mothers [15]. It is not necessarily a given that these repercussions would extend to youth’s fathers, as fathers and mothers respond differently to stress and relate differently to their children [19–23]. More generally, these results are aligned with research showing that criminal legal contact has symbiotic harm for family members of those caught up in the system [32].

Second, results show the association between youth police stops and fathers’ depression is concentrated among non-Black fathers and fathers of girls. Non-Black fathers (compared to Black fathers) and fathers of girls (compared to fathers of boys) are also less likely to have a youth who experiences a police stop [1]. This suggests that the unanticipated nature of police contact may be especially consequential, consistent with other research showing the spillover consequences of criminal legal contact are largest among those for whom it is unexpected [33]. These differential associations by race and sex are also consistent with research on the relationship between youth police stops and mothers’ mental health [14, 15]. Unanticipated stops—and the corresponding trauma, distress, and despair—may create a substantial shock to the broader family unit.

### Limitations

The Fragile Families data are the best available data to understand how fathers respond to youth police stops, but these data have limitations relevant to the analyses. First, the number of fathers who identify as primary caregivers at the 15-year survey is relatively small, which means that non-statistically significant results should be interpreted cautiously and that it is not possible to examine variation in stop experiences (e.g., intrusiveness) or variation across fathers’ pathways to being a primary caregiver. It is possible the association between youth police stops and fathers’ depression varies between fathers who have always been their youth’s primary caregiver and fathers who recently became primary caregivers (perhaps due to familial challenges such as maternal incarceration). Second, and relatedly, fathers’ depression was only ascertained when fathers identified as primary caregivers. The association between youth police stops and mental health among non-primary caregiver fathers remains an open question for future research. Third, the data preclude an examination of other indicators of mental health, and it is possible that the antecedents of fathers’ depression are different from the antecedents of other mental health conditions such as anxiety, and this is an additional question for future research. Fourth, fathers only reported the focal child’s police contact. They may have other children who experienced police contact and, if so, the estimate of the association between youth police stops and fathers’ depression is likely conservative. Finally, these data are not designed to estimate causal relationships between youth police stops and fathers’ mental health and, given youth police stops and fathers’ mental health were both measured at the 15-year survey (and fathers’ mental health is a measure of depression in the past year), it is possible that fathers’ depression preceded the youth being stopped. The results are robust to adjusting for a lagged dependent variable, which accounts for time-invariant characteristics of fathers, and placebo regression models suggest unobserved heterogeneity does not threaten causal inference. Future surveys should collect longitudinal information to facilitate analyses that can better account for time-varying characteristics that could lead to a spurious relationship between youth police contact and fathers’ depression.

## Conclusions

Youth police contact is experienced by more than one-quarter of youth born in urban areas [1]. It is also unequally distributed across the population such that youth of color are more commonly exposed to police contact than their counterparts [1]. Research increasingly documents the consequences of police stops for youth themselves, but the family-level spillover consequences of youth police stops are just beginning to be uncovered [15]. This research provides the first systematic accounting of how youth’s fathers experience spillover consequences of criminal legal contact. This focus on the experiences of men augments research on the consequences of men’s exposure to criminal legal contact and research on the consequences of this exposure for the children of men [34]. It also highlights how the intergenerational consequences of criminal legal contact can go from children to parents (as opposed to simply from parents to children). Given the consequences of men’s mental health for their own relationships, employment, and criminal legal contact and for the well-being of their children [25, 26], these repercussions of youth police contact may extend to other domains.
